# Comparison of Radiosensitization by HDAC Inhibitors CUDC-101 and SAHA in Pancreatic Cancer Cells

**DOI:** 10.3390/ijms20133259

**Published:** 2019-07-02

**Authors:** Simone Moertl, Sarah Payer, Rosemarie Kell, Klaudia Winkler, Natasa Anastasov, Michael J. Atkinson

**Affiliations:** 1 Helmholtz Center Munich, German Research Center for Environmental Health, Institute of Radiation Biology, 85764 Neuherberg, Germany; 2 Chair of Radiation Biology, Technical University Munich, 80333 Munich, Germany

**Keywords:** HDAC inhibitor, pancreatic cancer, ionizing radiation, apoptosis

## Abstract

Pancreatic cancer has a poor prognosis. New treatment options are urgently required to improve patient outcomes. One promising new class of anticancer drugs are synthetic histone deacetylase inhibitors (HDACi) which modulate chromatin structure and gene expression by blocking histone deacetylation. In this study, we aimed at comparing the in vitro capacities of the HDACi SAHA and CUDC-101 to increase radiosensitivity of human pancreatic tumor cell lines. Therefore, three pancreatic cancer cell lines (Su.86.86, MIA Paca-2, T3M-4) were treated with SAHA (1.5–5 µM) or CUDC-101 (0.25–3 µM) and after 24 h irradiated. Cell proliferation, clonogenic survival and apoptosis was determined. Additionally, cell lysates were investigated for the expression of apoptosis-related proteins. CUDC-101 and SAHA increased the radiation sensitivity of pancreatic tumor cell lines in a dose-dependent manner. This was evidenced by cell proliferation and clonogenic survival. Furthermore, enhanced radiation sensitivity after CUDC-101 or SAHA treatment was confirmed for Su.86.86 and T3M-4 cells in a 3-D microtissue approach. Increased amounts of subG1 cells and diminished full length PARP-1 suggest increased radiation-induced apoptosis after SAHA or CUDC-101 treatment. The comparison of both inhibitors in these assays manifested CUDC-101 as more potent radiosensitizer than SAHA. In line, western blot quantification of the apoptosis-inhibitory proteins XIAP and survivin showed a stronger down-regulation in response to CUDC-101 treatment than after SAHA application. These proteins may contribute to the synergy between HDAC inhibition and radiation response. In conclusion, these preclinical results suggest that treatment with the HDAC inhibitors CUDC-101 or SAHA can enhance radiation-induced cytotoxicity in human pancreatic cells. However, comparison of both inhibitors identified the multi target inhibitor CUDC-101 as more potent radiosensitizer than the HDAC inhibitor SAHA.

## 1. Introduction

Pancreatic cancer is one of the most lethal cancers worldwide with a 5-year survival rate under 5% and a median survival of 8–12 months [1,2]. At present, chemotherapy (mostly with the nucleoside analog gemcitabine) and radiotherapy in combination or alone are the standard treatments [3]. Since pancreatic cancer frequently develops therapy resistance, new agents and treatment modalities that increase tumor sensitivity and overcome drug resistance are needed [4].

Histone deacetylase inhibitors (HDACi) are anti-cancer drugs targeting protein acetylation [5,6]. Protein acetylation and deacetylation is exactly controlled by the competing activities of histone acetyl transferases (HAT) and histone deacetylases (HDAC). Histone acetylation leads to relaxation of chromatin associated with increased transcription of coding and non-coding RNAs, while deacetylation promotes chromatin condensation and transcriptional silencing. Beside histones, HDACs can also regulate the activity and metabolism of other proteins and substrates, to further broaden their biological impact [7,8]. By these means, HDAC inhibitors exert multiple cellular effects including cell cycle arrest, DNA repair, proliferation, autophagy, reactive oxygen species generation, apoptosis and angiogenesis [6].

First, HDACi were approved by the FDA as anticancer drugs and others were investigated in clinical trails, however resistance to HDACi often limits the success of these therapies [9]. This is especially the case in solid tumors, including pancreatic cancer [10]. To enhance the efficacy of HDACi in cancer therapy, combinations of treatments have been investigated. In vitro and in vivo experiments using various cancer cells have demonstrated that a combination of HDAC inhibitors with other anticancer drugs can produce synergistic or additive effects [11,12]. Regarding pancreatic cancer, preclinical studies showed increased anti-tumor activities for several HDAC inhibitors in combination with other chemotherapeutics [13,14,15].

In combination with ionizing radiation, HDACi have shown to be efficient radiosensitizers in several in vitro studies, with potent effects on prostate, glioma, melanoma, non-small cell lung cancer NSCLC, colon, squamous, osteosarcoma and lung cancer cell lines among many others [16].

The aim of this preclinical study was to examine the cell killing effect of the HDAC inhibitors CUDC-101 and SAHA in combination with radiation in pancreatic adenocarcinoma (PDAC) cancer cell lines.

SAHA is described as a pan-HDAC inhibitor blocking class I and class II histone deacetylases [17,18]. CUDC-101 was characterized as a multimodal inhibitor blocking simultaneously HDACs, epidermal growth factor receptor tyrosine kinase (EGFR/ErbB1), and human epidermal growth factor receptor 2 tyrosine kinase (HER2/neu or ErbB2) with potential antineoplastic activity in several breast and lung cancer cell lines [19,20]. A comparison of the inhibitory profiles of both substances is presented in Figure S1A.

## 2. Results

### 2.1. HDAC Inhibitors CUDC-101 and SAHA Increase the Radiation Sensitivity of Pancreatic Cancer Cells

To identify appropriate inhibitor concentrations MIA PaCa-2, Su.86.86 and T3M-4 cells were treated either with SAHA or CUCD-101 in a concentration range from 0,25 µM to 10 µM. 72 h after exposure cell viability was determined. IC50 values were approximately 4 µM for SAHA and 1 µM for CUDC-101 for each of the cell lines MIA PaCa-2, Su.86.86 and T3M-4 (Figure S2).

To test the effects of the inhibitors in combination with ionizing radiation cell numbers and clonogenic survival were measured using the sub IC50 inhibitor concentrations of 1.5 and 0.5 µM for SAHA and CUDC-101 respectively. For both concentrations a successful inhibition of HDACs was confirmed by increased amounts of the Ac-histone H3 and Ac-tubulin (Figure S1B,C). For CUDC-101 an inhibitory effect on EGFR/HER2 signaling was shown by reduced p-ERK1/2 (Figure S1B).

**Figure 1:**
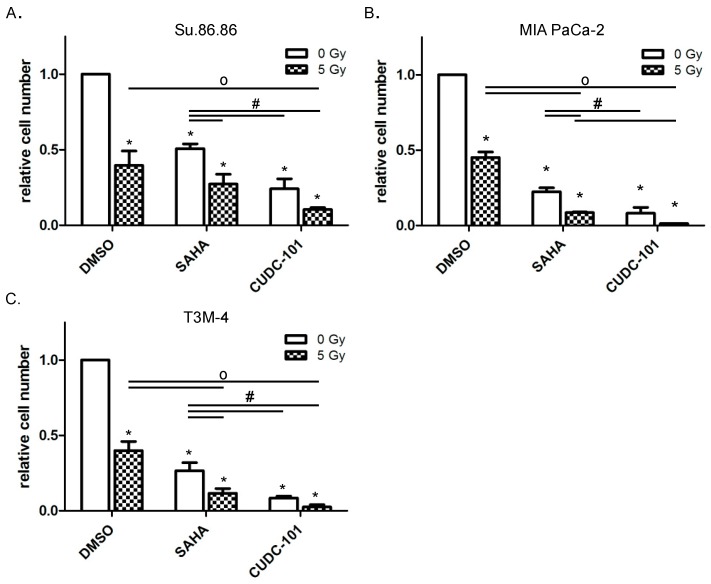
CUDC-101 more efficiently blocked proliferation than SAHA. Pancreatic cancer cell lines Su.86.86 (**A**), MIA Paca-2 (**B**) and T3M-4 (**C**) cells were treated with SAHA (1.5 µM) or CUDC-101 (0.5 µM) for 24 h, then cells were irradiated with 5 Gy or sham irradiated. After another 72 h cells were harvested and cell numbers were determined by counting. Histograms show the mean ± SEM of three independent experiments. For statistical analysis ANOVA was performed with post hoc Bonferroni multiple comparisons. * Indicates comparison with DMSO control, *p* < 0.01; # indicates comparison between SAHA and CUDC-101, *p* < 0.05; o indicates comparison between irradiated samples, *p* < 0.05.

**Figure 2:**
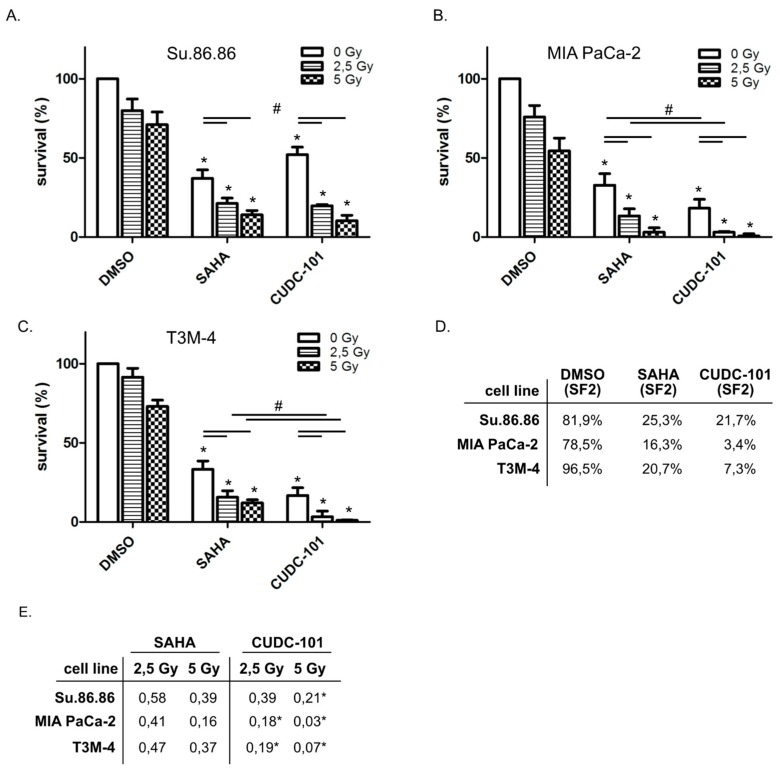
Clonogenic survival decreased more efficiently by combined CUDC-101 and radiation than by SAHA and radiation. Su.86.86 (**A**), MIA PaCa-2 (**B**) and T3M-4 cells (**C**) were treated with SAHA (1.5 µM) or CUDC-101 (0.5 µM) for 24 h, then cells were irradiated with 0, 2.5 or 5 Gy. After 10 days colonies bigger than 50 cells were counted. The mean of three independent experiments ± SD is shown. For statistical analysis, ANOVA was performed with post hoc Bonferroni multiple comparisons. * Indicates comparison with DMSO control, p < 0.05; # indicates comparison between SAHA and CUDC-101, *p* < 0.05. (**D**) Survival fractions after irradiation with 2 Gy. (**E**) Mean *n*-fold changes between inhibitor treatment compared to combined inhibitor/radiation treatment, * indicates comparison between SAHA and CUDC-101 treatment at the indicated dose, *p* < 0.05).

In both cases, the treatment with compound alone and in combination with radiation significantly decreased cell counts (Figure 1) and survival (Figure 2). From Figure 1 it is visible that cell numbers were reduced to 50% or below compared to dimethylsulfoxide (DMSO) treated controls by SAHA or CUDC-101. An additional radiation exposure during HDACi treatment further reduced cell numbers in all three cell lines. The comparison of cell counts in SAHA and CUDC-101 treated samples showed a more potent effect for CUDC-101 in all three cell lines.

To further investigate the interaction of HDACi CUDC-101 and SAHA with radiation, the colony forming ability of the pancreatic cancer cell lines was tested (Figure 2A–C). Both HDAC inhibitors as well as radiation alone reduced clonogenic survival. The combined treatment of radiation exposure and SAHA or CUDC-101 induced a further reduction. The determination of the survival fraction at 2 Gy (SF2) again showed the more potent effect of CUDC-101 in MIA PaCa-2 and T3M-4. In Su.86.86 at least similar SF2 fractions were achieved with a three-fold lower concentration of CUDC-101 than with SAHA (Figure 2D). Moreover, as seen in the proliferation assay Su.86.86 seems to be more resistant than MIA PaCa-2 and T3M-4 cells.

### 2.2. 3D Microtissue Growth Is Delayed More Efficiently by Combined HDAC Inhibitor and Radiation Treatment

**Figure 3:**
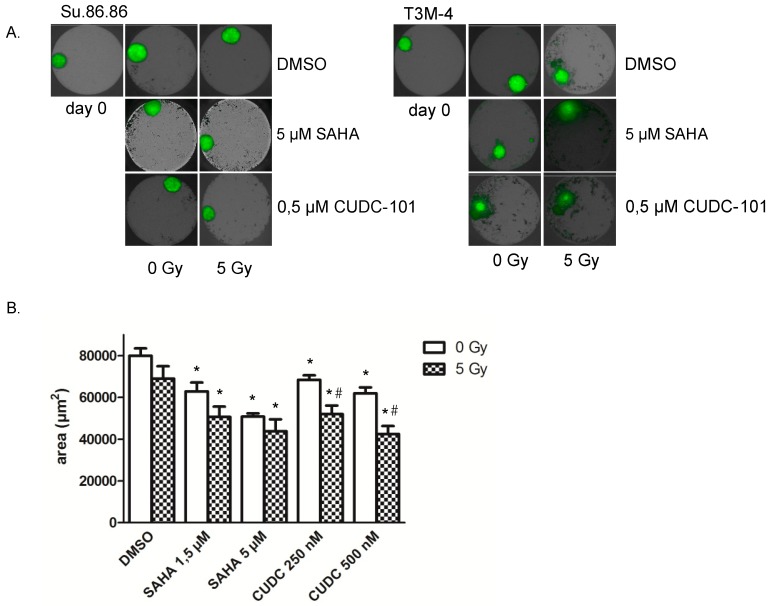
(**A**) Growth of 3D-microtissues was delayed after HDAC inhibitor and radiation treatment. Microtissues were grown from Su.86.86 (left) and T3M-4 (right) cells for 3 days, after that SAHA (1.5 and 5 µM) and CUDC-101 (0.25 and 0.5 µM) were added, another 24 h later cells were irradiated with 0 or 5 Gy. After 6 days pictures were taken with the high content screening system Operetta. (**B**) Areas of Su.86.86 microtissues were determined with the Operetta system. Data represent the mean of at least three independent experiment ± SD. For statistical analysis, ANOVA was performed with post hoc Bonferroni multiple comparisons. * Indicates comparison with DMSO control, *p* < 0.01, # indicates comparison between irradiated and non-irradiated CUDC-101 samples *p* < 0.05.

Su.86.86 and T3M-4 cells were able to form 3D microtissues (Figure 3A). MIA PaCa-2 cells did not form 3D microtissues under the applied conditions. Irradiation with 5 Gy alone showed a weak effect on microtissue growth, while SAHA and CUDC-101 delayed the growth in a dose-dependent manner in Su.86.86 cells (Figure 3B). An additional delay was observed with the combined exposure to HDAC inhibitor CUDC-101, while the reduction induced by a combination of SAHA and radiation was not significant. Comparison of both inhibitor treatments suggested a more potent effect of CUDC-101 as similar growth delays were achieved at lower inhibitor concentrations. In T3M-4 cells SAHA, CUDC-101 and irradiation all individually induced a growth delay. Here this was associated with the disassembly of microtissues (Figure 3A, right). For the CUDC-101 treatment, an enhanced effect between inhibitor treatment and radiation exposure was visible compared to the treatments alone. Together combined effects of inhibitor treatment and radiation were evident in 3D which confirmed 2D cell culture data.

### 2.3. HDAC Inhibitors Promote Radiation-Induced Apoptosis

**Figure 4:**
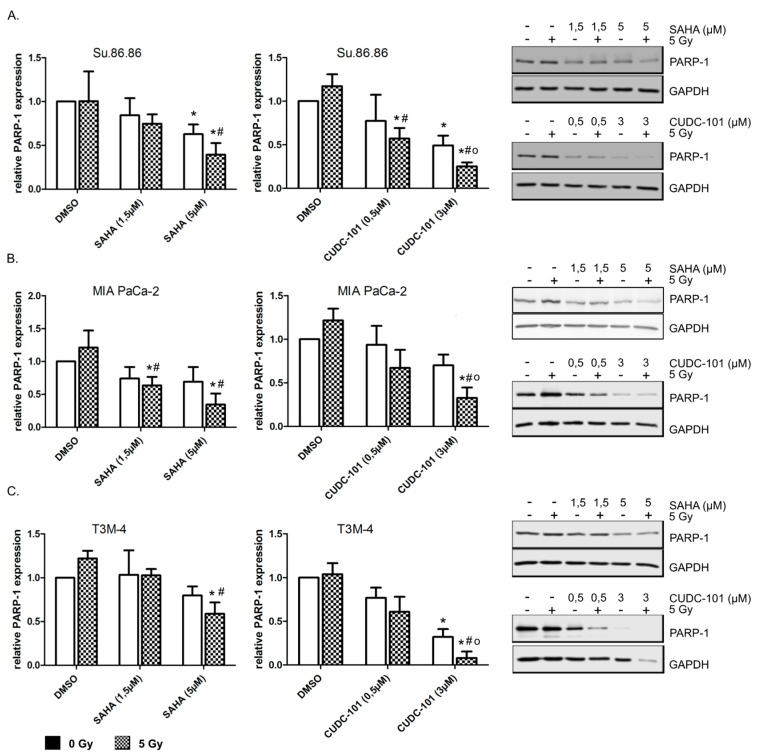
Radiation promoted CUDC-101 induced PARP-1 reduction. Su.86.86 (**A**), MIA PaCa-2 (**B**) and T3M-4 (**C**) cells were treated with SAHA or CUDC-101 for 24 h, then cells were irradiated with 5 Gy. 24 h after irradiation full length PARP-1 was quantified by western blot. (Left) relative PARP-1 amounts normalized to GAPDH and control amounts. Data represent the mean of at least three independent experiment ± SD. For statistical analysis ANOVA was performed with post hoc Bonferroni multiple comparisons. * Indicates comparison with DMSO control, *p* < 0.01, # indicates comparison with DMSO, 5 Gy, *p* < 0.05; o indicates comparison with CUDC-101, 0 Gy, *p* < 0.05. (right) representative western blots.

In order to identify the mechanism of SAHA or CUDC-101-triggered radiosensitivity, DNA repair and apoptosis was quantified. As visible in Figure 4, irradiation alone showed a tendency to increase the DNA repair protein PARP-1, while SAHA or CUDC-101 treatment decreased the expression (Figure 4). The reductions caused by CUDC-101 were more pronounced by the combined action of the inhibitor and irradiation and also more pronounced than the reductions observed after SAHA treatment. However, the amount of residual DNA double strand repair foci 24 h after treatment was not affected by the inhibitor treatments alone or in combination with radiation (Figure S3).

**Figure 5:**
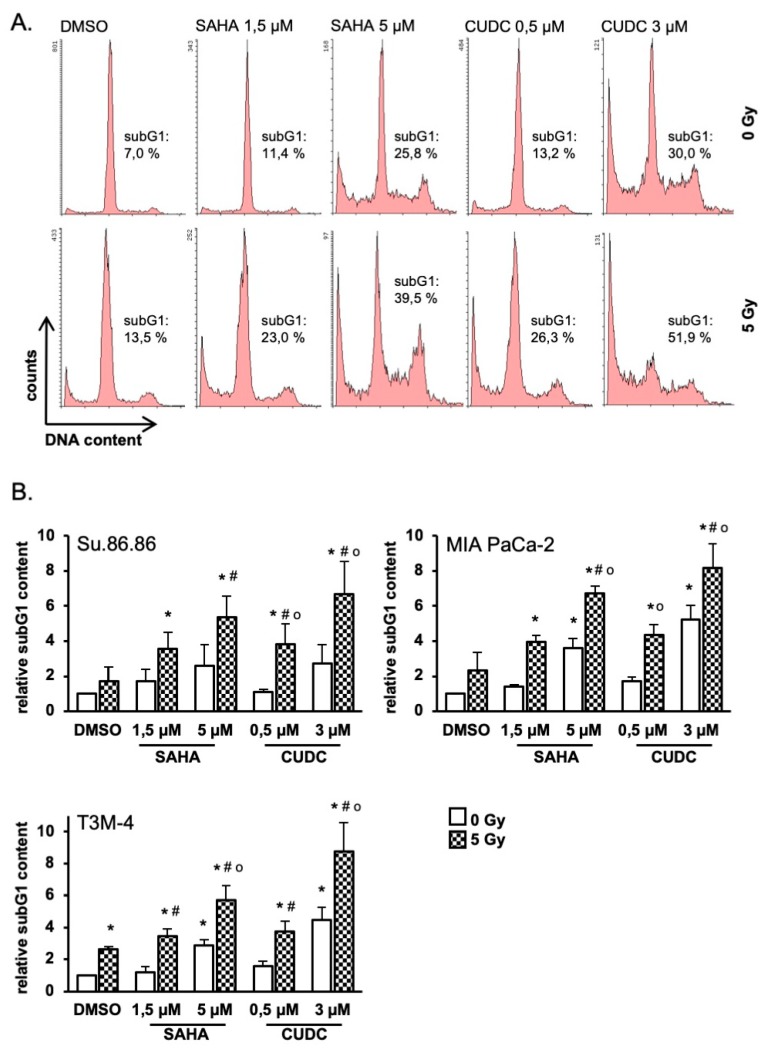
CUDC-101 increased radiation-induced apoptosis more than SAHA and IR. Su.86.86, MIA PaCa-2 and T3M-4 cells were treated with SAHA (1.5 µM) or CUDC-101 (0.5 µM) for 24 h, then cells were irradiated with 0, 2.5 or 5 Gy. (**A**) 72 h after irradiation apoptotic cells were quantified as subG1 population by FACS, MIA PaCa-2 is shown as a representative example. (**B**) SubG1 content 72 h after irradiation relative to non-irradiated control cells. Data represent the mean of at least three independent experiment ± SD. For statistical analysis ANOVA was performed with post hoc Bonferroni multiple comparisons. * Indicates comparison with DMSO control, *p* < 0.01, # indicates comparison between SAHA and CUDC-101 *p* < 0.05; o indicates comparison with DMSO, 5 Gy, *p* < 0.05.

Quantification of subG1 cells as an indicator of apoptosis showed modest increases in all three cell lines after irradiation or HDAC inhibitor exposure alone (Figure 5). For both inhibitors increases of subG1 population were concentration-dependent. Significantly more subG1 phase cells were detected after combined treatment of radiation and SAHA or CUDC-101 compared to compound or radiation alone. MIA PaCa-2 and T3M-4 cells were more sensitive compared to Su.86.86 cells. In line with cell proliferation and clonogenic survival CUDC-101 was more potent than SAHA as comparable subG1 contents were induced by lower inhibitor concentrations.

### 2.4. Prosurvival Factors XIAP and Survivin Are Reduced after Combined CUDC-101 and Radiation Treatment

To further assess the mechanism of radiation, CUDC-101 and SAHA triggered apoptosis, the expression of 35 apoptosis-related proteins was quantified. Reverse phase arrays were performed from pooled lysates obtained from three biological replicates per treatment and *n*-fold changes compared to DMSO/0Gy controls were calculated (Table S1).

We were able to detect several proteins uniformly regulated in all three cell lines. Radiation alone triggered the expression of the antiapoptotic protein Bcl-2 and the apoptosis regulator protein Bcl-x in all three systems. After the HDAC inhibitor treatment, these changes were less consistent. After exposure to CUDC-101 or SAHA and radiation, increased cleaved caspase-3 levels indicated increased apoptosis. Also decreased levels of claspin in CUDC-101 or SAHA in irradiation-treated cells support higher apoptosis. Decreased amounts of the apoptosis-inhibiting proteins XIAP and survivin were detected in most of the samples treated with SAHA or CUDC-101 alone or in combination with radiation.

**Figure 6:**
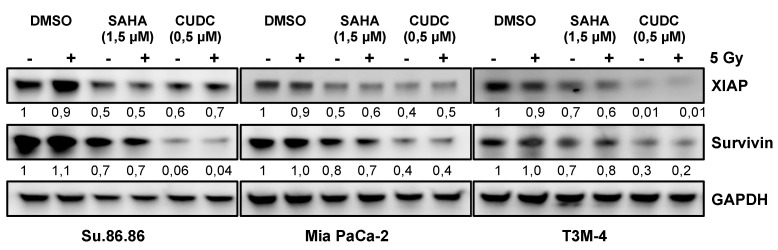
Expression of antiapoptotic factors XIAP and survivin is reduced after combined HDAC inhibitor/radiation treatment. A representative western blot (out of 3 biological replicates) of survivin and XIAP protein expression after treatment with CUDC-1 and/or irradiation. *n*-Fold change compared to non-irradiated control is indicated. + irradiated with 5 Gy, − non-irradiated

To validate the deregulation of XIAP and survivin after compound or compound/irradiation treatment, both proteins were quantified by western blot in further biological replicates. XIAP expression was not changed by radiation alone, while SAHA or CUDC-101 treatment reduced the protein amount in all three cell lines. A more pronounced reduction was observed for CUDC-101 in T3M-4. The combined radiation and compound treatment yielded no further reduction on the expression of XIAP in our cells. In addition, survivin was not changed by radiation alone, while SAHA or CUDC-101 treatment reduced the protein amount. However, in all three cell lines CUDC-101 induced a stronger reduction of survivin compared to SAHA, even at a lower concentration (Figure 6).

## 3. Discussion

Pancreatic cancer is a highly malignant tumor with a general resistance against radiotherapy. HDACi have been shown to be effective anticancer agents in other tissues [21]. However, their full therapeutic potential is only achieved in combination with other agents and / or radiation [22,23]. The current study compared the efficacy of HDACi SAHA or CUDC-101 in combination with ionizing radiation against pancreatic tumor cells.

### 3.1. SAHA and CUDC-1 Both Increase Radiosensitivity of Pancreatic Cancer Cells

Exposure to either SAHA or CUDC-101 prior to IR (ionizing radiation) decreased the proliferation of all three tested pancreatic cell lines. Additional irradiation further reduced the cell numbers indicating the higher efficiency of a combined treatment. Clonogenic survival was also decreased under these conditions. A synergistic action of HDAC inhibitors and IR was further supported by a reduced 3D microtissue growth after combined treatment compared to HDACi or IR alone. The comparison of the effects of the HDAC inhibitors on cell numbers and clonogenic survival indicate that CUDC-101 was more efficient than SAHA alone or with irradiation. A CUDC-101 concentration of 0.5 µM leads to equal or even higher sensitization than SAHA in a 3-fold higher concentration.

SAHA is one of the well-established HDACi and has been shown to act as a radiosensitizer in vitro and in vivo in different tumor entities, including pancreatic cancer [24,25,26]. CUDC-101 is described as a multitarget inhibitor against HDACs, EGFR, and HER2, but the exact mechanism of action is presently unknown [19,20]. Therefore, the more potent cell killing of pancreatic cancer cells may be a result of the parallel targeting of HDACs and receptor tyrosine kinases.

The recent literature assigned the radiosensitizing effect of HDAC inhibitors to defects in the DNA damage signaling and the double strand break repair pathways homologous recombination and non-homologous endjoining [6,16]. Although the reduced levels of the DNA repair protein PARP-1 would be in line with these findings, our data on γ-H2AX foci did not support impaired double strand break repair as reason for the increased radiosensitivity in our cell lines at the used concentrations. In none of the three test systems the combined treatment of HDAC and IR resulted in an increase in non-repaired DNA breaks 24 h after irradiation (Figure S3), however this finding did not exclude disturbances in repair kinetics or repair pathway choice. Additionally, DNA breaks arising from apoptosis may interfere with radiation-induced breaks.

### 3.2. IR Increases the Apoptotic Response to SAHA and CUDC-101 Treatment

HDACi mediated tumor cell death is often shown to be due to an induction of apoptosis, which occurs through both, intrinsic or extrinsic pathways, leading to caspase-3 activation and cell death [5,27]. Moreover, defects in apoptotic pathways and the deregulation of apoptotic proteins, such as Bcl-2, Bcl-xL and Mcl-1, play decisive roles in the development and therapy resistance of pancreatic cancer [28].

Our data showed increased active caspase-3 and subG1 population which suggests increased apoptotic cell death as underlying mechanism for the observed radiosensitizing effect of SAHA or CUDC-101 treatment. As PARP-1 is a direct target for cleavage by caspase-3, the reduced levels of the protein can be interpreted as further evidence of augmented apoptosis. Increased apoptosis is in line with our results obtained from proliferation and clonogenic survival and further validates the synergistic action of SAHA, or CUDC-101 plus IR.

### 3.3. Reduced XIAP and Survivin May Account for Reduced Survival

The analysis of apoptosis-related proteins identified the downregulation of XIAP and survivin after exposure to CUDC-101 or SAHA. Exposure to the HDAC inhibitors plus IR has no further significant effect.

Both proteins are members of the Inhibitor of apoptosis protein (IAP) family. Synergistically they inhibit apoptosis by targeting caspase-3 and -9 and induce resistance to conventional chemotherapeutic agents in various cancer cells [29]. Recent data also demonstrated that a knockdown of survivin or XIAP leads to increased radiosensitivity in various cancer cells, including pancreatic cancer [30,31,32] as well as in normal tissue [33]. Previous studies have shown that XIAP and survivin were overexpressed in pancreatic cancer and were closely associated with cell proliferation and chemoresistance to the standard chemotherapeutic gemcitabine [34]. Additionally, they are both acknowledged as radioresistance factor with potential as a predictive molecular marker for metastases and cancer patient survival following radiation therapy [35,36].

According to these findings we suggest that CUDC-101 or SAHA trigger a reduction of the antiapoptotic proteins XIAP and survivin which leads then to increased radiation sensitivity through enhanced apoptosis. However, more a pronounced reduction of survivin by CUDC-101 than by SAHA may contribute to the more efficient radiosensitizing.

Mechanistically both protein reductions may be explained by a HDAC inhibitor induced reduction of mutated p53. Mutated p53 constitutively activates NFκB which is a transcription factor of XIAP and survivin [37]. On the other hand, mutated p53 abundance in pancreatic cancer cells depends on the activity of HDAC1, HDAC2 [38,39]. As SAHA and CUDC-101 block both activities and all of our cell lines harbor p53 mutations it is very likely that a HDAC inhibitor induced p53 reduction causes less NFκB activity resulting in lower amounts of XIAP and survivin. This suggestion is substantiated by reduced p53 protein amounts after inhibitor treatment in our experimental setup (Figure S2A). But as survivin expression is lower after CUDC-101 than after SAHA treatment while p53 is similar after both inhibitors we suggest further regulatory mechanisms after CUDC-101 treatment (Figure 6 and Figure S2B). Supported by recent findings which showed a synergy between EGFR/HER2 blocking and SAHA treatment in MiaPaca-2 cells [40] we propose the inhibitory function of CUDC-101 on receptor tyrosine kinase signaling as such mechanism.

In conclusion, our study indicates that a combination of the HDAC inhibitors SAHA or CUDC-101 with ionizing radiation was more effective in the killing of pancreatic cancer cells than the individual treatments. CUDC-101 was the more potent radiosensitizer compared to SAHA. As increasing therapy response is a key factor for improving pancreatic cancer prognosis, this study provided evidence that CUDC-101 combined with radiation could be used as a successful combination therapy for pancreatic cancer in the future.

## 4. Material and Methods

### 4.1. Cell Lines and Irradiation

The human pancreatic adenocarcinoma cell lines Su.86.86 (p53G245S) [41], T3M-4 (p53Y220C) [42] and MIA PaCa-2 (p53R248W) [43] were used. Su.86.86 and T3M-4 cells were cultivated in RPMI 1640 (Gibco, Waltham, MA, USA) with Glutamax™-I and 10% FCS. MIA PaCa-2 cells were cultivated in DMEM high glucose medium with GlutaMAX™-I, 4.5 g/L glucose, pyruvate and 10 % FCS. All cell lines were cultivated at 37 °C in 5 % CO_2_. Su.86.86-GFP and T3M-4-GFP cells (expressing green fluorescent protein) were established by lentiviral transduction using pGreenPuro transfer vector (SBI, Palo Alto, CA, USA) as previously described [44]. For stable and constitutive GFP expression cells were cultivated in medium containing 0.3 µg/mL puromycin.

Cell line identification was confirmed by Eurofins Genomics (Ebersberg, Germany) by sequencing of nine different loci: D5S818, D13S317, D7S820, D16S539, VWA, TH01, AM, TPOX, CSF1PO. Mycoplasma negative status was confirmed with MycoAlert (Lonza, Basel, Switzerland).

Cells were irradiated using an X-Strahl RS225 with a 3.0 mm aluminium filter with 195 kV/10 mA X-rays at a dose rate of 0.82 Gy per min.

### 4.2. HDAC Inhibitors

CUDC-101 (Cat. #S1194, 99.4% purity, Selleckchem, Munich, Germany) and SAHA (Cat. #S1047, Selleckchem, Munich, Germany) were dissolved in DMSO, prepared at a stock concentration of 10 mM and stored at −20 °C. All inhibitor and control treatments resulted in a 1% DMSO concentration in the cell culture medium.

### 4.3. Immunoblot Analysis

Cells were lysed in lysis buffer II (120 mM NaCl, 25 mM Tris-HCl, pH 7.5, 1% Triton X-100) supplemented with 1 mM protease inhibitors (sodium orthovanadate and phenylmethanesulfonyl fluoride, Roche, Germany) for 60 min on ice. For histone analysis 1 × 10^6^ cells were incubated in TEB buffer (0.5% Triton X-100, 2mM PMSF in PBS) for 10 min on ice and then centrifuged (2000rpm, 10 min). Cell pellets were washed in 50 µL TEB buffer and then incubated in 50 µl 0,2N HCl at 4 °C overnight. After centrifugation (2000 rpm, 10 min) histone lysates were stored at −20 °C. Western blot analysis was performed according to standard procedures using enhanced chemiluminescence detection (Amersham ECL Select Western Blotting Detection Reagent, GE Healthcare, Chicago, IL, USA). For detection of the proteins primary antibodies directed against PARP-1 (ab32138, Abcam, Milton, Cambridge, USA), survivin (ab76424, Abcam, Milton, CA, USA), XIAP (sc-55551, Santa Cruz Biotechnology, Dallas, TX, USA), ERK1/2 (#9102, Cell Signaling, Danvers, MA, USA), p-ERK1/2 (#9101, Cell Signaling, Danvers, MA, USA), acetyl-tubulin (sc-23950, Santa Cruz Biotechnology, Dallas, TX, USA) and acetyl-histone H3 (#9677, Cell Signaling, Danvers, MA, USA) were used. Protein loading was monitored by detection of GAPDH (sc-47724, Santa Cruz Biotechnology, Dallas, TX, USA), histone H3 (#4499, Cell Signaling, Danvers, MA, USA) or tubulin (sc-32293, Santa Cruz Biotechnology, Dallas, TX, USA). As secondary antibodies goat anti-mouse IgG-HRP (sc-2005, Santa Cruz Biotechnology, Dallas, TX, USA) and goat anti-rabbit IgG-HRP (sc-2004, Santa Cruz Biotechnology, Dallas, TX, USA) were used. For quantification of images TotalLab TL 100 software (TotalLab, Newcastle, UK) was used.

### 4.4. Cell Proliferation

To quantify cell proliferation 2 × 10^4^ cells were seeded in 2 mL growth medium in 6-well plates in duplicates. 24 h after seeding of the cells HDAC inhibitors were added and after another 24 h cells were irradiated. 72 h after irradiation cell numbers were counted in a Coulter Counter (Beckman Coulter, Krefeld, Germany).

### 4.5. Cell Viability

Cell viability after HDAC inhibitor treatment was measured with the Presto Blue assay (Thermo Fisher Scientific, Munich, Germany). Therefore 7.500 cells were seeded in 96-well plates in 100 µL medium. 24 h later HDAC inhibitors were added in the indicated concentrations. After another 72 h 10 µL PrestoBlue™ Cell Viability Reagent was added and cells were incubated for 30 min at 37 °C. Fluorescence was measured in a plate reader (TECAN, Maennedorf, Switzerland) at 560 nm excitation and 590 nm emission.

### 4.6. Clonogenic Survival

To measure clonogenic, survival cells were seeded in 6-well plates. After 24 h HDAC inhibitors were added and another 24 h later cells were treated with 2.5, 5 and 10 Gy. Then the cells were incubated for at least 9 days to allow colony formation from single cells. Subsequently cells were washed twice with PBS, fixed with 100 % ethanol (30 min) and finally stained with Giemsa solution (1:20 in PBS, 30 min) (Boehringer Ingelheim, Ingelheim, Germany). Excessive dye was removed and colonies with more than 50 cells were counted by eye. SF2 fractions were calculated according to the linear quadratic model using S(D) = e^–(αD + βD2)^ with D (dose) and S (surviving fraction).

### 4.7. 3D Microtissue Analyses

Individual 3D microtissues were generated by plating 2000 cells per well in gravity trap ultra-low adhesion 96-plates (Perkin Elmer, Waltham, MA, USA), containing 100 µL growth medium. After 3 days (defined as day 0 of treatment) formed microtissues were incubated with inhibitors at the indicated concentrations. The microtissues were irradiated 24 h later. Controls were treated with equal volumes of DMSO. Growth of eight spheroids per treatment was monitored after 10 days using a high content screening system Operetta (Perkin Elmer) and the maximal area of GFP expressing microtissue (μm^2^) was determined using the Harmony analysis Software (Perkin Elmer) as previously reported [45].

### 4.8. SubG1 Analysis

Cell cycle distribution was analyzed 48 h after irradiation by determining the DNA content using propidium iodide (PI) staining and flow cytometry. Cells were harvested and collected by centrifugation at 300 g and 4 °C for 5 min. The cell pellet was washed twice with PBS and either stored at −20 °C till staining or immediately processed. Cell pellets were resuspended in Cell Cycle Solution (1 g/L NaCitrate, 500 µL/L Triton X-100, 10 mg/L RNase A (bovine pancreas), 50 µg/mL PI) as previously described [46]. Cell cycle distribution was analyzed using a FACScan LSR II (excitation wavelength: 488 nm, emission wavelength: 610 nm; BD Biosciences) and Flowing Software version 2.5.1 (Perttu Terho, Turku Centre for Biotechnology, University of Turku, Finland). Cells with a DNA content lower than cells in G1 phase were defined as the subG1 fraction and were considered to be apoptotic.

### 4.9. γ-H2AX Foci

For γ-H2AX focus analysis, 3 × 10^5^ cells were seeded on slides in 4-well chambers and treated with SAHA or CUDC-101. 24 h later cells were irradiated. Another 24 h later cells were fixed in 4% paraformaldehyde and permeabilized with 0.2% Triton X-100 in PBS for 5 min followed by blocking with 1% BSA for 60 min. The cells were probed with anti-gamma-H2AX (Ser139) antibody (dilution 1:250, cat. # 05-636, Millipore, Darmstadt, Germany) overnight at 4 °C followed by a 1 h incubation at room temperature in the dark with fluorescence-labeled secondary antibody (dilution 1:500, Cy-3 goat anti mouse IgG (cat. #016-160-084, Jackson ImmunoResearchLab, Cambridgeshire, UK). Nuclear counterstaining was performed with Hoechst 33342 (Sigma, Munich, Germany) and cover slips were mounted with Vectashield (Vector Laboratories, Burlingame, CA, USA). Analysis was performed with the fluorescence microscope Biorevo BZ-9000 (Keyence, Osaka, Japan). The number of residual foci 24 h after irradiation was determined in 100 nuclei per condition.

### 4.10. Human Apoptosis Protein Array

A human apoptosis array containing 35 different antibodies (Proteome Profiler™ Ary009; R&D Systems, Wiesbaden, Germany) was used to analyze apoptosis-related protein profiles according to manufacturer instructions. In brief, the total cell lysates (250 µg pooled from three biological replicates) were first incubated with the array membrane overnight at 4 °C, followed by incubation with a biotinylated detection antibody cocktail at room temperature for 1 h. A digital imaging system (Alpha Innotec, Kasendorf, Germany) was used to detect the chemiluminescent signals, which were further analyzed using the ImageJ program.

### 4.11. Statistical Analysis

Data are presented in figures as means of biological replicates ± SD or SEM as indicated. Significance analysis was done by analysis of variance (ANOVA) followed by post hoc Bonferroni testing for multiple comparisons. Statistical significance was defined as a *p* value ≤ 0.05.

## Supplementary Materials

Supplementary materials can be found at https://www.mdpi.com/1422-0067/20/13/3259/s1.
