# Supplementary material for: Comparison of Radiosensitization by HDAC Inhibitors CUDC-101 and SAHA in Pancreatic Cancer Cells
Source: Int J Mol Sci. 2019 Jul 2;20(13):3259. doi: 10.3390/ijms20133259 (PMC6651299; doi:10.3390/ijms20133259)
Supplement: Supplementary file 1 [file ijms-20-03259-s001.zip › ijms-525855/SupFig1.pdf]

A.

| | class I HDAC | | | | class II HDAC | | | | | | | Ref. |
|---|---|---|---|---|---|---|---|---|---|---|---|---|
| compound | 1 | 2 | 3 | 8 | 4 | 5 | 6 | 7 | 9 | 10 | | |
| SAHA | +++ | + | ++ | + | o | o | +++ | o | o | ++ | | [18, 39] |
| CUDC-101 | +++ | ++ | +++ | + | ++ | ++ | +++ | + | ++ | ++ | EGFR, HER2 | [19] |

activity of inhibition: o = low, + = moderate, ++ = good, +++ = high

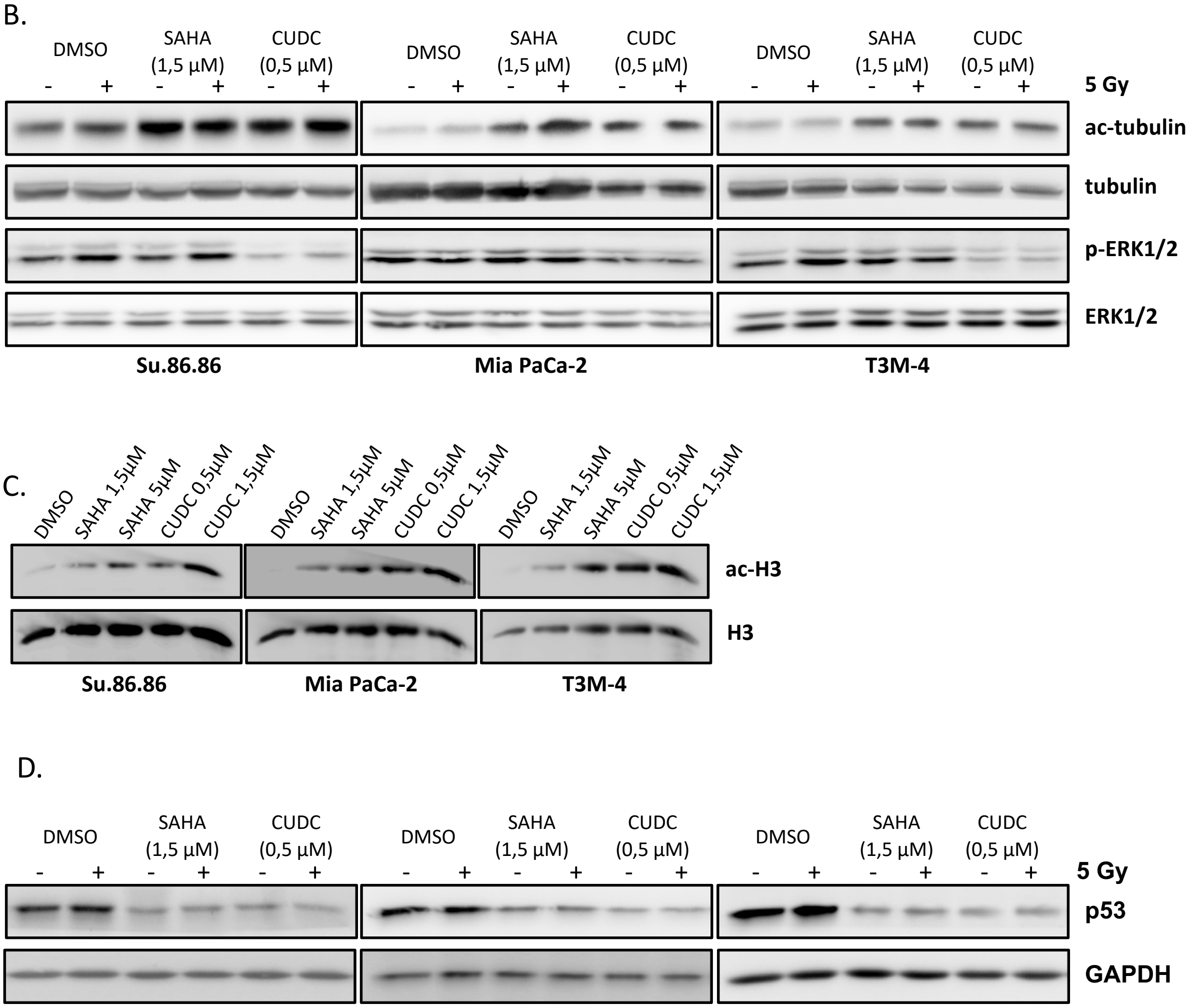


**Supplementary Figure 1: SAHA and CUDC-101 activities in pancreatic cancer cells** (A) summary of published activities of SAHA and CUDC-101 on histone deacetylases (B) representative western blot of SAHA and CUDC-101 effects in whole cell lysates obtained from Su.86.86, MiaPaca-2 and T3M-4 cells (n=2) (C) western blot of histone lysates of SAHA or CUDC-101 treated pancreatic cancer cells (n=1) (D) representative western blots showing SAHA or CUDC-101 effects on p53 protein expression (n=2).
