# Supplementary material for: Comparison of Radiosensitization by HDAC Inhibitors CUDC-101 and SAHA in Pancreatic Cancer Cells
Source: Int J Mol Sci. 2019 Jul 2;20(13):3259. doi: 10.3390/ijms20133259 (PMC6651299; doi:10.3390/ijms20133259)
Supplement: Supplementary file 1 [file ijms-20-03259-s001.zip › ijms-525855/SupFig2.pdf]

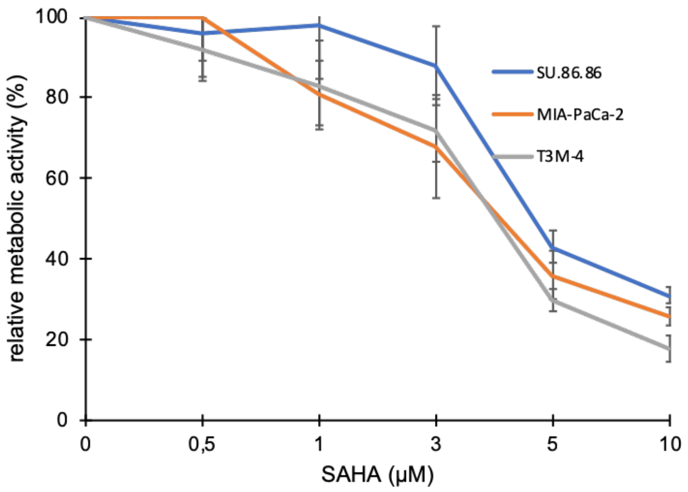

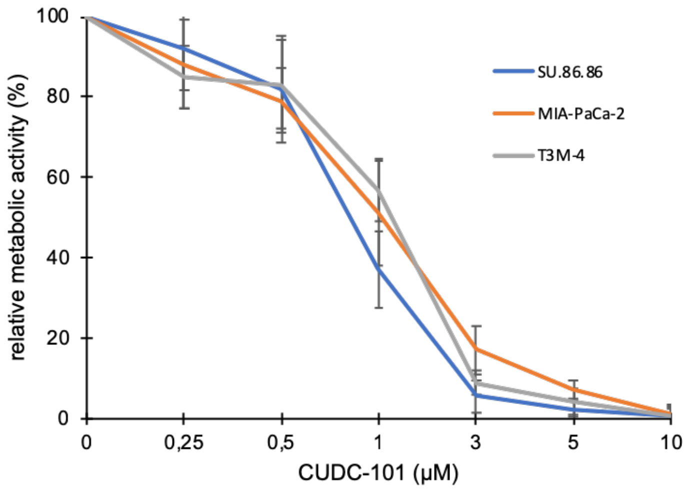

Supplementary Figure 2: Sensitivity of pancreatic cancer cells to HDAC inhibitor treatment. Viability of cell lines Su.86.86, MIA PaCa-2 and T3M-4 72 h after SAHA or CUDC-101 treatment relative to DMSO controls (measured by Presto Blue assay). Data represent the mean of three independent experiment +- SD.
