# Supplementary material for: Comparison of Radiosensitization by HDAC Inhibitors CUDC-101 and SAHA in Pancreatic Cancer Cells
Source: Int J Mol Sci. 2019 Jul 2;20(13):3259. doi: 10.3390/ijms20133259 (PMC6651299; doi:10.3390/ijms20133259)
Supplement: Supplementary file 1 [file ijms-20-03259-s001.zip › ijms-525855/SupFig3.pdf]

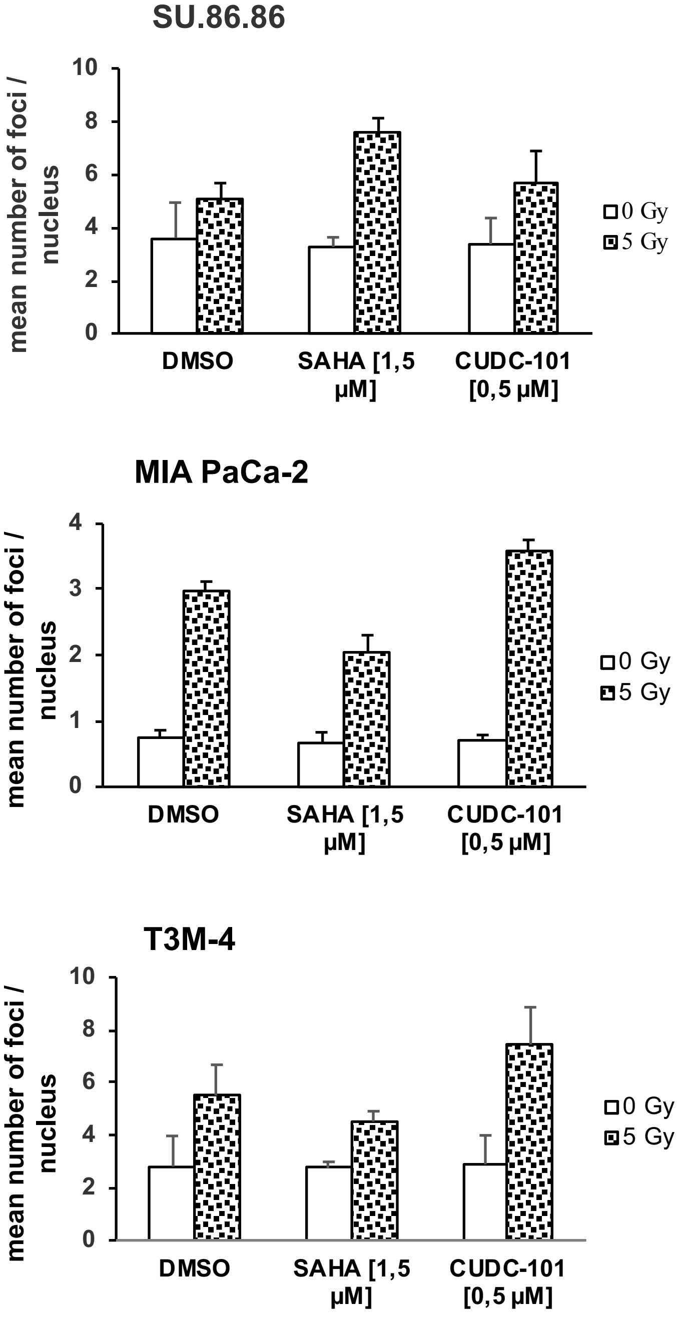

Supplementary Figure 3: Gamma-H2AX foci in pancreatic cell lines after treatment with SAHA or CUDC-101 with/without irradiation. SU.86.86, MIA PaCa-2 and T3M-4 cells were treated with SAHA (2 µM) or CUDC-101 (1 µM). 24 h later cells were irradiated with 5 Gy. After a repair time of 24 h gamma-H2AX was detected by immunostaining and fluorescence microscopy (foci in 100 nuclei were counted). Data represent the mean number of foci per nucleus obtained from three independent experiments ± SD.
